# Upper Airway Control Therapy (U‐ACT): The Development of a Non‐Pharmacological Intervention for Inducible Laryngeal Obstruction

**DOI:** 10.1111/resp.70073

**Published:** 2025-06-10

**Authors:** Jemma Haines, Jaclyn A. Smith, Stephen J. Fowler, Janelle Yorke

**Affiliations:** ^1^ Faculty of Biology, Medicine and Health, School of Biological Sciences, Division of Infection, Immunity & Respiratory Medicine The University of Manchester Manchester UK; ^2^ NIHR‐Manchester Biomedical Research Centre, Manchester University NHS Foundation Trust Manchester UK; ^3^ Faculty of Health and Social Sciences, School of Nursing, The Hong Kong Polytechnic University Hung Hom Hong Kong

**Keywords:** behaviour therapy, behavioural change techniques, complex intervention, inducible laryngeal obstruction, non‐pharmacological, vocal cord dysfunction

## Abstract

**Background and Objective:**

Non‐pharmacological intervention is the recommended gold standard for inducible laryngeal obstruction (ILO) treatment. Despite this, there is no standardised approach and interventions are poorly described. The objective was to develop and describe a standardised non‐pharmacological intervention for ILO, for future testing of effectiveness.

**Methods:**

MRC guidelines for complex intervention development were followed; the methodological approach was structured using the INDEX principles. The multi‐phase research stages were: (1) evidence review; (2) qualitative data collection from speech and language therapists (*n* = 7) and patients (*n* = 22); (3) intervention design and theoretical underpinning; (4) prototype survey feedback from Stage 2 participants; and (5) final intervention description, using a validated reporting framework.

**Results:**

Systematic review and synthesis of 14 studies (*n* = 527) identified key uncertainties and steered Stage 2 interviews. Framework analysis of qualitative data collected identified five overarching key themes for inclusion. The resulting ‘Upper Airway Control Therapy’ (U‐ACT) intervention comprises two core components (education & empowerment; reliever breath control), four supporting components (bio‐feedback training; prevention methods; supporting co‐existing conditions; managing others' reactions to ILO) and a cross‐cutting home practice component. U‐ACT's mechanisms of action to bring about change includes 36 behaviour change techniques. Feedback on U‐ACT protype was extremely positive; survey responders (*n* = 23; 87% response rate) strongly agreed to acceptability statements for all parameters surveyed [5‐point Likert scale; median (range), 4.5, 3–5].

**Conclusion:**

The U‐ACT intervention, developed with keystakeholders and underpinned with a programme theory, is fully manualised and ready for evaluation. If future testing proves clinical and cost effectiveness, it could be incorporated into existing ILO services.

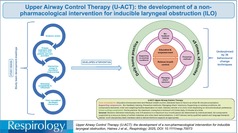

## Graphical abstract

The developed ‘Upper Airway Control Therapy’(U‐ACT) manualised intervention describes a standardised non‐pharmacological behavioural therapy intervention for inducible laryngeal obstruction. U‐ACT is systematically and robustly developed with key‐stake holders and is underpinned with programme theory. If proven clinically and cost effective, U‐ACT will provide robust evidence for best practice management guidelines.

**See related** editorial

## 1 Introduction

Inducible laryngeal obstruction (ILO) is a debilitating condition causing breathing difficulties, due to inappropriate laryngeal obstruction at the glottic and/or supraglottic level [1]. Individuals typically report transient inspiratory problems with acute onset and tightness localised to the throat, often in response to an aggravating trigger [2]. Presentation ranges from mild to acute respiratory distress [3]. Complexity arises as ILO can occur in isolation, mimic other disorders, or act as a multimorbidity with other diseases [4]. Although evidence on the mechanistic drivers contributing to ILO remains absent, there is international consensus ILO is a multi‐factorial, complex condition, with no single overarching mechanism [4]. This offers some explanation why many individuals with ILO report high levels of morbidity impacting quality of life [5] and the condition creates a challenge to healthcare systems [6].

Once an ILO diagnosis is objectively confirmed, due to the paucity of data, there is poor understanding of what the most effective ILO treatment is [7]. No recognised standardised treatment approach exists, but non‐pharmacological therapy‐based management is commonly referred to as the beneficial gold standard [4, 8]. In the clinical context, this is often delivered by speech and language therapists (SLTs). This empirically established role is well grounded in a wide body of research and clinical evidence in the functional laryngology space [9, 10] and underpinned by SLTs' trained expertise in the management of a broad spectrum of laryngeal disorders (e.g., dysphonia [11], dysphagia [12], chronic cough [13]). However, despite a non‐pharmacological approach being the commonly cited treatment, efficacy is largely unknown. Systematic reviews highlight preliminary support [7, 14] but studies are mainly low‐level evidence with a high risk of bias, thus precluding true insight into their effectiveness [15]. Without robust evidence, non‐pharmacological ILO interventions cannot be promoted as best practice and may explain current heterogeneity in care.

In essence, non‐pharmacological ILO interventions utilise behavioural techniques to address symptoms and modify maladaptive laryngeal postures [16]. Pragmatically, the primary goal is to specifically teach control of the laryngeal area and maintain an adequately open airway during respiration. In practice, this involves delivery of multi‐modal interacting components in an attempt to effect change and therefore can be considered a complex intervention [17, 18]. Clinician judgements, based on patients' predisposing and perpetuating factors, typically leads to individualised treatment. However, commonality of components is evident [19] even though the specific characteristics of interventions are poorly described. The ‘active’ ingredients and mechanisms of change are rarely defined, which is a common issue in behavioural change interventions; between 36% and 89% of interventions that seek to change health behaviours not being clearly theory based [20]. Outcome reporting often focuses on specific symptom endpoints [15], that are consequences of a behaviour rather than the behaviour itself. Such outcome‐focused evidence provides little information about how treatment works, and the therapeutic processes that are responsible for producing change [21]. A preliminary study [22] investigated potential key behaviour change techniques [23] (BCTs) across three data sources relating to speech therapy non‐pharmacological ILO interventions (literature review; semi‐structured interview with a care giver; observations of six intervention sessions). Across all three data sources forty‐seven BCTs were identified and six (e.g., reduced negative emotions) were present in all three sources. This provides useful insight into the potential salient BCTs used in non‐pharmacological interventions and the potential mediators of change, but the work was small scale meaning findings are limited.

When considering a standardised approach, there are many key uncertainties in the delivery of non‐pharmacological interventions used to treat adults with ILO; the components of intervention, the underlying processes that support them, acceptability and mode of delivery are yet to be explored. Further, how any intervention may interact with the context in which it is delivered is unknown. Investigation and development of a standardised intervention is therefore warranted, and the UK Medical Research Council (MRC) provides well‐established guidance on approach [17, 18, 24]. Further, it is important in any intervention development that there is completeness of reporting to ultimately improve replicability and support uptake, which is often slow and sometimes negligible [25]; in a review of non‐pharmacological intervention studies (*n* = 80), only 29% were adequately described [26]. The goal of this work was therefore to develop a non‐pharmacological ILO intervention, guided by the MRC framework [18], ready for future feasibility testing. Specifically, we aimed to develop and comprehensively describe, in line with the ‘Template for intervention description and replication’ (TIDieR) framework [27], a well‐defined, reproducible, standardised non‐pharmacological intervention for ILO that will be supportive of patient need.

## 2 Methods

### 2.1 Overview of the Development Process

**FIGURE 1:**
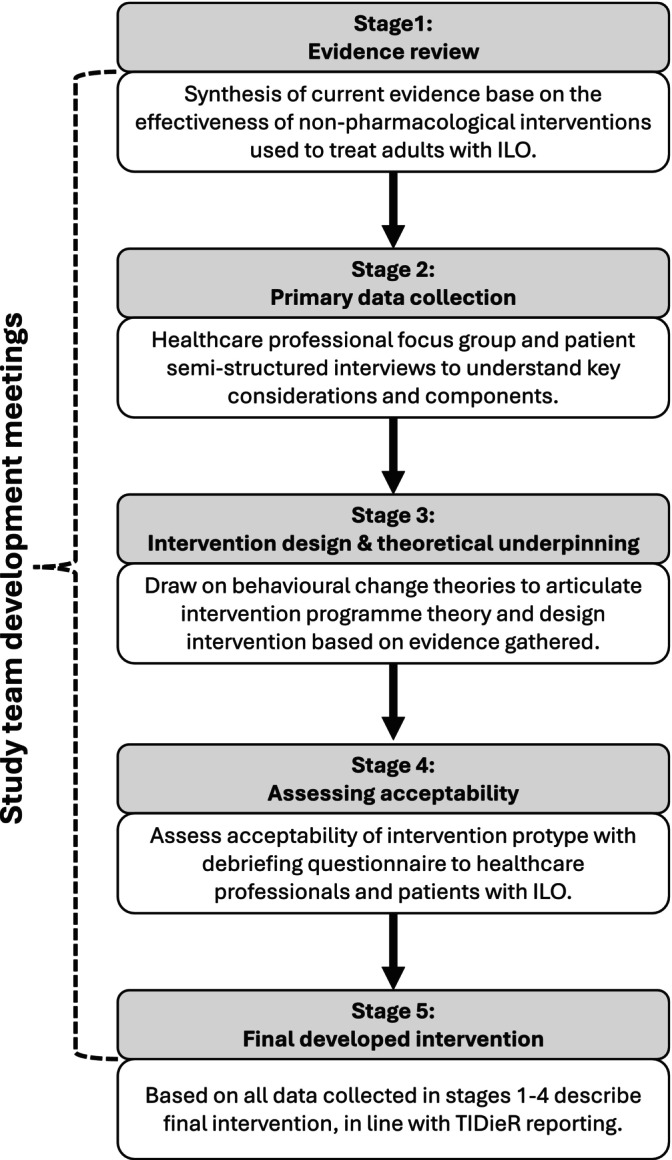
Intervention development process.

The MRC framework for developing and evaluating complex interventions [18] guided the methodological approach, supported by the INDEX principles for intervention development and design [28]. The multi‐phase research steps (Figure 1) structured the intervention development process and ensured key‐stakeholder engagement and identification of an underpinning programme theory. Study team development meetings occurred regularly throughout the duration of the study to support research integrity and enable discussion of findings to facilitate iterative development. The study team comprised representation of professions involved in ILO management (speech therapy, respiratory medicine and nursing). The TIDieR framework [27] was followed to report and describe the final intervention. Intervention development focused on an adult population due to major anatomical differences between paediatric and adult larynges [29] and the differing approaches to clinical care management. Further, ILO induced by exercise was not considered as evidence suggests it has an alternative underlying pathophysiological mechanism when compared to ILO not induced by exercise [30], meaning treatment targets may differ. Study approval and registrations were obtained (Northwest Greater Manchester South Research Ethics Committee 24/NW/0010; ISRCTN18291587; PROSPERO CRD42020213187). Informed consent was obtained from all study participants. Work was conducted between January 2021 and December 2024.

### 2.2 Stage 1: Evidence Review

A systematic review of the literature occurred to identify existing non‐pharmacological interventions used to treat adults with ILO. We have previously reported the full methodological approach applied [15]. In summary, electronic databases (MEDLINE, EMBASE, CINAHL, PsycINFO, AMED, CENTRAL) were systematically searched. A PICO framework was applied to identify studies [31] of adults with ILO (population) who had received non‐pharmacological treatment (intervention) with or without a control intervention (comparison) and where the effectiveness of these interventions was considered (outcome). Two reviewers independently screened a representative sample, with lead‐author completion due to excellent inter‐rater reliability. Data was extracted using a pre‐defined piloted form. Methodological quality was appraised (blindly by two reviewers) using the JBI Critical Appraisal Tools [32]. A narrative synthesis was performed due to the heterogeneity of studies.

### 2.3 Stage 2: Primary Data Collection

To identify key considerations and components for a standardised non‐pharmacological ILO intervention, qualitative data were collected prospectively during a focus group and semi‐structured interviews with key stakeholders; we have previously reported the full methodological steps [33]. Participants included SLTs (*n* = 7), intervention naïve patients (*n* = 6) and patients who had received a non‐pharmacological intervention targeting ILO (*n* = 16). Interview guides were structured using the COM‐B (capability, opportunity, motivation, behaviour) model for understanding behaviours [34], and questions addressed the key uncertainties identified in Stage 1 evidence review. A framework method thematic analysis [35, 36] was conducted to explore and identify themes in the data.

### 2.4 Stage 3: Intervention Design and Theoretical Underpinning

Using the data from Stage 1 and 2, BCTs were identified utilising the BCT Taxonomy Version 1 (BCTTv1) list of 93 hierarchically organised BCTs [37], and the TIDieR framework [27] structured the approach. This identified the smallest active components designed to change behaviour in the developing intervention. The identified BCTs were then mapped to relevant intervention functions and behavioural change theory, specifically the behavioural change wheel (BCW) [34], to inform the intervention's mechanisms of action. Numerous behaviour change theories exist, but the overarching BCW was selected as it synthesises 19 frameworks of behaviour change into a coherent model. Specifically, the BCW core ‘hub’ layer, the COM‐B model, was beneficial as it recognises behaviour is part of an interacting system involving ‘capability’, ‘opportunity’ and ‘motivation’. In developing an intervention, it is important to understand which/all combinations of these need targeting to bring about change and therefore guided a theoretical base on which to develop our intervention. The application of the BCW and COM‐B model is recommended to facilitate systematic application of theory and evidence to complex intervention design [38] and has been used in over 150 peer‐reviewed publications relating to intervention development [34]. Finally, the BCTs were refined using the Theory and Techniques Tool (https://theoryandtechniquetool.humanbehaviourchange.org/tool). This prioritised the BCTs that have shown a strong link with mechanisms of action for the relevant COM‐B elements identified in our evidence.

Iterative development of a supporting programme of theory began at study outset. The introductory version was guided by the study team's extensive clinical experience in the field (cumulative > 85 years) and existing evidence base. During the programme of study, it was adapted and refined (based on data collected and BCT mapping) to a resulting version that portrayed the theoretical basis for the described intervention. Within the programme theory, context detail and economic considerations were included (core elements of the 2021 MRC framework). A visual representation summarising the final intervention programme theory was presented in a causal logical model. This enabled illustration of how the differing components related to one another, considering context and detailing the mechanisms of action.

### 2.5 Stage 4: Assessing Acceptability

The developed intervention protype and a customised de‐briefing questionnaire were shared with Stage 2 participants [SLTs(*n* = 7) and patients who had received a non‐pharmacological intervention targeting ILO (*n* = 16)]. The debriefing questionnaire was administered electronically via the REDCap web survey platform; questions were structured in line with the TIDieR [27] framework to inform on all aspects of the intervention protype. It measured attitudes towards the intervention using a 5‐point ordinal (Likert) scale, to rate if respondents agreed or disagreed with a statement [39] (e.g., “I think the name and description of the intervention are acceptable”). These were analysed quantitatively, using the median to demonstrate the measure of central tendency and support interpretation of results [40]. Free text responses were reviewed and any themes identified were reported to supplement understanding on acceptability.

### 2.6 Stage 5: Final Developed Intervention

The final developed intervention was manualised and described using the TIDieR framework [27]. The intervention design and reporting included: an intervention name; intervention rationale; required materials and procedures; who should provide; how the intervention is delivered; where and when it is delivered; dosing; and finally, specific details on tailoring options. This enabled description in enough detail to support replication.

## 3 Results

### 3.1 Evidence Synthesis Findings

We have previously fully reported the systematic review results [15]. Key findings are summarised in Table S1.

### 3.2 The Views of Speech and Language Therapists and Patients

The framework thematic analysis results are fully reported in our associated publication [33]. Five main themes were identified as the key considerations and components for a standardised non‐pharmacological ILO intervention. These were: (i) ‘Living with ILO’ (highlighted rationale and motivation); (ii) ‘Pathway to intervention’ (offered understanding on the wider context related to access); (iii) ‘Care delivery’ (answered important key uncertainties relating to intervention implementation); (iv) ‘Intervention content’ (detailed specific components); and (v) ‘Views of interventions’ (identified mediators of change). Table S2 provides a synthesis of findings to give content grounding for reporting this overall programme of study.

### 3.3 Identified Behavioural Change Techniques and Their Mechanisms of Action

Analysis of Stage 1 and 2 data identified 36 unique BCTs (Table 1), across 14 of the 16 BCTTv1 categories. All BCW intervention functions were included, except coercion (creating an expectation of punishment or cost). The COM‐B behavioural diagnosis identified presence in all aspects of the model, with dominance for psychological capability and reflective motivation.

**TABLE 1:** Behavioural change techniques mapped to intervention functions and behaviour change theory.

Intervention components based on evidence gathered	Behaviour change technique(s)	Behavioural change wheel intervention function(s)	Behaviour change theory: COM‐B behavioural diagnosis
***Naming and description***			
Remove speech and language therapy name	✓ Framing/reframing	Education	Reflective motivation
***Materials***			
General written resource explaining ILO and developed intervention	✓ Credible source	Education Enablement	Reflective motivation
Written and video bank resource for individuals receiving intervention	✓ Demonstration of the behaviour ✓ Behavioural practice & rehearsal ✓ Habit formation	Modelling Training	Physical capability Social opportunity Reflective motivation
***Procedures/components***			
Education about ILO	✓ Re‐attribution ✓ Reduce negative emotions ✓ Goal setting (outcome) ✓ Verbal persuasion about capability ✓ Information about others' approval	Education Persuasion	Psychological capability Reflective motivation
Reliever breath control techniques (escalating and acute ILO episodes)	✓ Problem solving ✓ Behavioural substitution ✓ Habit reversal ✓ Generalisation of a target behaviour ✓ Self‐talk	Education Enablement Training	Psychological & physical capability Automatic & reflective motivation
Managing others' reactions to ILO	✓ Remove aversive stimulus ✓ Social support (practical) ✓ Information about others' approval	Enablement Environmental restructuring	Psychological capability Physical & social opportunity
Visualisation of laryngeal movements in real time using laryngoscopy	✓ Biofeedback ✓ Satiation ✓ Exposure	Education Training	Psychological & physical capability Physical opportunity
Prevention methods (e.g., diaphragmatic breathing)	✓ Instruction on how to perform a behaviour ✓ Information about antecedents ✓ Demonstration of the behaviour ✓ Body changes ✓ Feedback on outcome(s) of behaviour	Environmental restructuring Education training Modelling Restriction	Psychological & physical capability Reflective motivation
Supporting co‐existing conditions	✓ Pharmacological support ✓ Reduce negative emotions	Education Enablement	Physical opportunity
Home practice/application of techniques	✓ Self‐monitoring of outcomes of behaviour ✓ Behaviour practice & rehearsal ✓ Habit formation ✓ Prompts/cues ✓ Graded tasks ✓ Problem solving ✓ Action planning ✓ Discrepancy between current behaviour and goal ✓ Commitment ✓ Monitoring of emotional consequences ✓ Incentive (outcome)	Enablement Incentivisation Modelling	Psychological & physical capability Physical opportunity Reflective motivation
***Provider***			
Trained, knowledgeable and skilled in treating ILO (likely a speech and language therapist)	✓ Credible source	Enablement	Reflective & automatic motivation
***How, where, when and how much, tailoring***			
Delivered individually, coordinated with other members of care team Personalised to needs of individual (feedback/delivery mode/dosing)	✓ Goal setting (behaviour) ✓ Problem solving ✓ Review behaviour goal(s) ✓ Verbal persuasion about capability (if appropriate) ✓ Feedback on behaviour	Environmental restructuring Enablement	Physical & social opportunity Psychological capability

### 3.4 Structure of Developed Intervention

Specific intervention components and the draft intervention structure were identified from the Stage 2 data analysis. A dedicated study team meeting discussed and agreed the final intervention prototype. Refinements included (i) adding ‘empowerment’ within the education component to capture patient data relating to the importance of addressing pre‐diagnosis fears and improve understanding of ILO (ii) altering home‐practice as a cross‐cutting component (not a separate component) in response to healthcare professional data indicating it as a ‘golden thread’ (iii) being prescriptive on home‐practice dosing to address key uncertainties previously identified.

**FIGURE 2:**
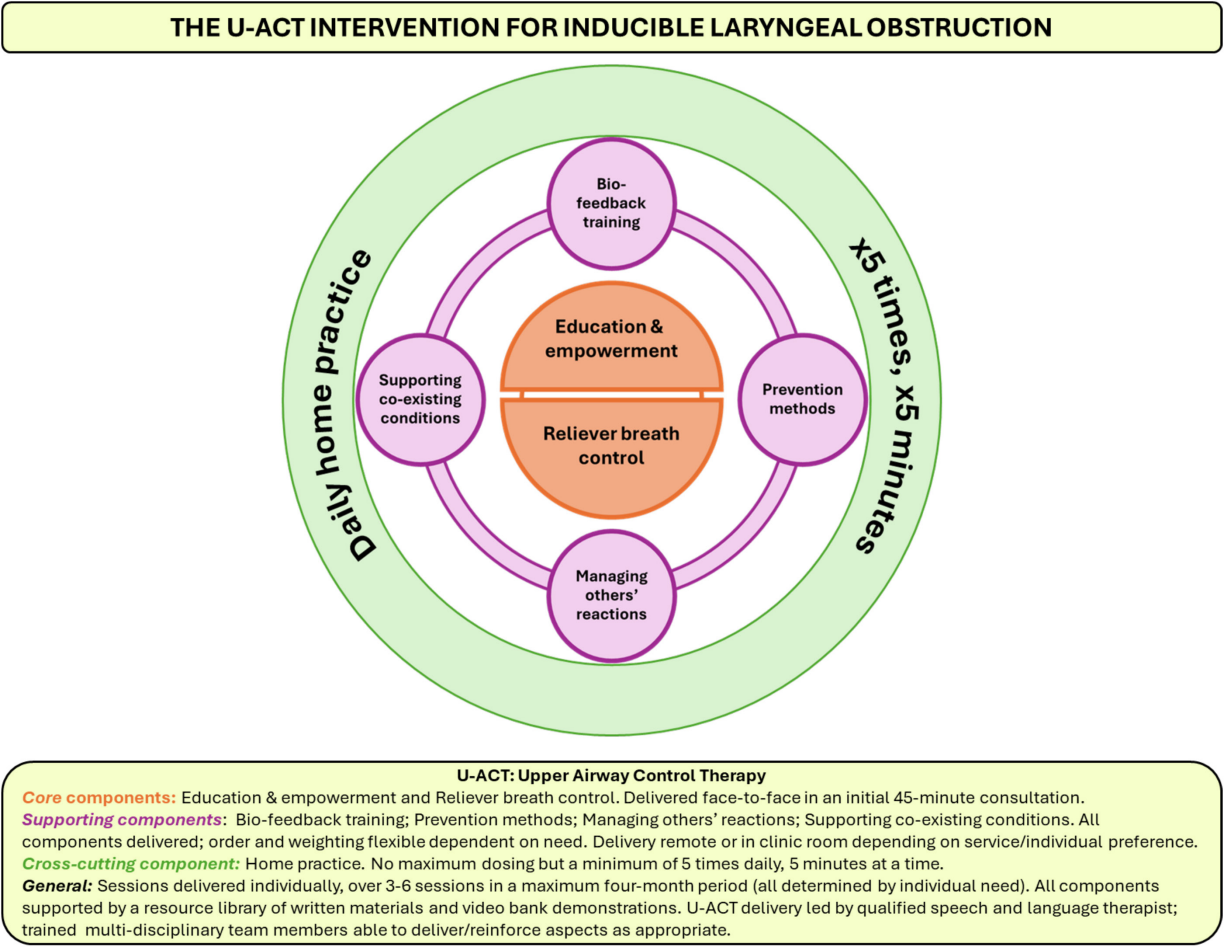
Structure of the U‐ACT intervention for inducible laryngeal obstruction.

**FIGURE 3:**
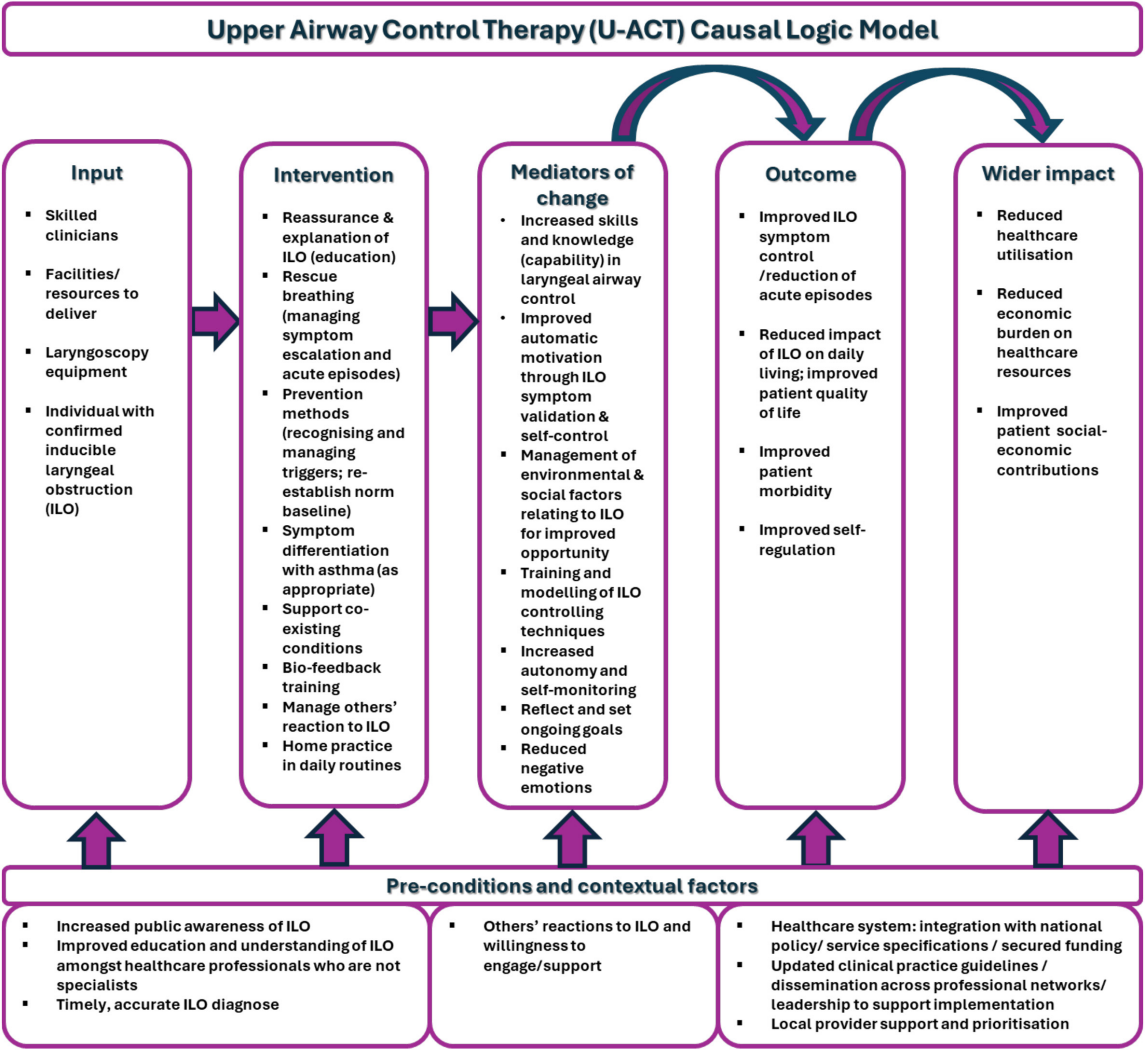
U‐ACT causal logic model.

The ‘Upper Airway Control Therapy’ (U‐ACT) intervention comprises two core components (education & empowerment; reliever breath control), four supporting components (bio‐feedback training; prevention methods; supporting co‐existing conditions; managing others' reactions to ILO) and a cross‐cutting home practice component (Figure 2). Intervention delivery occurs 1:1 over a maximum four‐month period. The initial face‐to‐face session comprises the core components and lasts 45 min. The further two to five sessions are 30 min in duration and flexibly cover all remaining components. These sessions can be delivered either face‐to‐face or remotely (virtual or telephone) and at any time point within the four‐months, depending on patient/service need and preference. The fully described intervention (Table 3), programme theory and accompanying causal logic model (Figure 3) are subsequently presented.

### 3.5 Programme Theory

The inaugural programme theory identified initial links between likely intervention components and proposed outcomes. The key‐stakeholder engagement and analysis of collected study data enabled refinement of contextual factors. Further, it provided specific detail on intervention activities and gave insight and evidence for the mechanisms of action to support outcome. This resulted in U‐ACT's developed programme theory, which is presented as a causal logical model in Figure 3.

### 3.6 Acceptability of Intervention Protype

Response to the debriefing questionnaire was excellent at a cumulative 87% response rate (healthcare professionals, 86%; patients, 88%), which was well above the global average response rates in patient and healthcare professional surveys (55% and 70%, respectively) [42]. There was overwhelming positivity for the developed intervention (Table 2), indicating intervention acceptability to both healthcare professionals and patients with ILO. Following analysis of results, only minor amendments to the final reported intervention occurred. These were (i) adding further description of intervention naming to make clearer U‐ACT is designed specifically for individuals with ILO and not those with other related upper airway disorders like refractory chronic cough, (ii) specific mention of psychological support and onward referral as necessary in the ‘supporting co‐existing conditions’ component, (iii) added description on training requirements for those delivering the intervention and (iv) inclusion of U‐ACT format and expectation in the ‘initial education and empowerment’ session.

**TABLE 2:** Debriefing questionnaire results.

Question	5‐point Likert scale agreement rating (1 = strongly disagree, 2 = disagree, 3 = neutral, 4 = agree, 5 = strongly agree); median (range)	Example additional comments or reasons for answers (HCP, healthcare professional; Pt, patient)
I think the name and description of the intervention are acceptable	4.5 (3–5)	“*I love the term control in the title*” [Pt] “*The title says exactly what it is trying to do – much better than speech therapy*” [Pt] “*Think needs to be a bit clearer for ILO and not cough control in description*” [HCP]
I think the rationale and goals of intervention are acceptable	5 (3–5)	“*Spot on*” [Pt] “*The rationale is super clear*” [HCP]
I think the described materials that will support the intervention are appropriate	5 (3–5)	“*I feel that it covers everything that I would have found beneficial. Even down to acknowledging how others (*i.e., *family & colleagues) reactions to episodes can have an impact*” [Pt] *“A video bank is a really good idea”* [Pt]
I think the activities described that will be used in the intervention are acceptable	4.5 (3–5)	*“I really like that home practice is a cross‐cutting component of the other six intervention components”* [Pt] *“Wondering if need to detail onward referral to psychology as needed somewhere?”* [HCP] *“I think it is really comprehensive and covers all key things”* [Pt] *“I really like how the intervention is broken down and what is covered in the sessions, and that it can be delivered flexibly”* [HCP]
I think who will deliver the intervention is acceptable	4 (3–5)	“*I would be interested to see more detail of the training; I think it might be useful to relate this to existing RCSLT guidance or competency frameworks”* [HCP] *“Agree speech therapists should be the primary deliverer of this intervention but MDT input to support therapy strategies is appropriate”* [HCP]
I think how the intervention will be delivered is acceptable	4 (3–5)	*“Face to face is essential for initial consultations/assessment but video/telephone are acceptable substitutes for follow‐up – I like how that is possible”* [HCP] *“Good that the first session is in person”* [Pt]
I think where the intervention will occur is acceptable	4.5 (3–5)	No comments received
I think the description of when and how much intervention will be given is acceptable	4 (3–5)	*“Great to have a guide of how much intervention may be offered, but with some flex depending on patient/clinical need”* [HCP] *“This seems ideal”* [Pt]
I think the description of how the intervention will be personalised is appropriate	4.5 (3–5)	*“It feels there is flexibility within the intervention for both patient and service variability—*that is, *it's not too rigid and prescriptive and can use clinical judgement to tweak how intervention is delivered when necessary”* [HCP] *“I like that ‘managing others’ is included as an option to tailor therapy. This could also be good to highlight as an option at the start of treatment, to normalise this as a part of the intervention model. I imagine that this could enhance engagement in therapy and enhance time to discharge”* [HCP]

*Overall general comments*
***“Having read the intervention manual, I felt heard and understood from my own experiences of living with ILO. I believe if this becomes the standardised process across all health trusts ILO sufferers and their families will benefit”* [Pt]** *“Being given all the tools and the support makes a huge difference. Having the video bank demonstrations available in one place will mean you have that extra support anytime and means you can take control and feel empowered as opposed to feeling alone”* [Pt] *“Thank you so much. This going forward will benefit so many other ILO sufferers, it will make such a difference”* [Pt] *“I think this will benefit ILO sufferers immensely”* [HCP] ***“This will be amazing for our service in the future thank you so much”* [HCP]**

### 3.7 The Final Developed Intervention

Table 3 describes the final developed U‐ACT intervention. The full U‐ACT handbook and training checklist guide is presented in Appendix S1. The handbook provides a detailed description of U‐ACT's components and related content. The specific tasks within the content lists are not overly prescribed to allow for clinician preference and flexibility based on individual patient need. For example, in the *Prevention methods* component ‘diaphragmatic breathing’ is listed content but specific techniques to achieve this are not detailed. In this case, the clinician could utilise Accent Method [43] foundations, a lying to standing hierarchy focusing on reduced clavicular movement, semi‐occluded vocal tract exercise [44] or any other direct exercise to achieve diaphragmatic control. Similarly, although all intervention components are delivered, flexibility for choosing intervention dosing, component weighting and order of delivery is based on individual need; some components may require review and reinforcement across several sessions. Such clinical decision making is within the intervention provider's gift. This requires a level of ILO clinical skill and autonomy; the training checklist guide supports ensuring this is evident.

**TABLE 3:** The U‐ACT intervention.

Name and brief description of intervention
Upper Airway Control Therapy (U‐ACT): A non‐pharmacological behavioural therapy intervention for adults with inducible laryngeal obstruction (ILO). The U‐ACT intervention is designed to give control of ILO symptoms and reduce the impact of ILO on adult individuals diagnosed with the condition.

Rationale and theory
Adults with ILO suffer significant impact on quality of life and often have high healthcare utilisation; there is frustration amongst healthcare professionals and patients about the lack of understanding for optimal ILO non‐pharmacological treatment methods. The U‐ACT intervention is developed to offer a standardised approach, provide clarity on intervention components and identify the underlying mechanisms that aim to achieve positive outcomes. The developed U‐ACT intervention comprises 36 behavioural change ‘active ingredient’ techniques. These provide individuals with psychological and physical capability to control ILO, the opportunity to perform techniques in differing environments and strengthened motivation to consciously goal plan, with added self‐belief that they can control ILO.

Materials
*U‐ACT promotional materials* Information regarding the U‐ACT intervention. Aimed for referrer clinicians, patients and associates, the wider multi‐disciplinary team and healthcare professionals. Includes general education about ILO. To provide: with healthcare provider correspondence following ILO diagnosis to individuals when diagnosed with ILO and referred for U‐ACT intervention as a general educational resource to improve awareness of ILO *Provider training materials* The U‐ACT handbook (Appendix 10) provides an information delivery manual and a training checklist for healthcare professionals who will deliver the intervention. *Patient information materials* A written resource to support each of the U‐ACT intervention components (see procedures). Provide relevant written resource following delivery of specific component (electronically or paper based depending on individual preference). A video bank resource, comprising all direct physical exercises (see procedures). Videos demonstrate intervention techniques and support the modelling and training function of U‐ACT intervention. Provide access following first intervention session. *Physical materials* Laryngoscopy imaging equipment with visibly accessible feedback monitors (see procedures, bio‐feedback training) U‐ACT intervention comprises two core components (education & empowerment; reliever breath control), four supporting components (bio‐feedback training; prevention methods; supporting co‐existing conditions; managing others' reactions to ILO) and a cross‐cutting home practice component. *Education and empowerment (core component)* Activity content supported by written informational resource and home practice. Content includes: Normal respiration and the larynx. Laryngeal movements during ILO. Reassurance and explanation ILO not life‐threatening (discuss ‘worst‐case’ scenario). Current scientific understanding on causes and curability of ILO (in accessible language), that is, limited to date and therefore focus is on symptom control. Self‐management approaches. Explain U‐ACT intervention development (i.e., informed by needs and experiences of individuals suffering with ILO) and discuss intervention structure to set expectations. Individual goal setting. Outcome monitoring. *Reliever breath control (core component)* Activity content supported by written informational resource, video bank demonstration and home practice. Content includes: Rescue breathing (focus on nasal inspiration and focused expiration, for example, sniff/blow technique). Managing ILO escalation through application of rescue breathing. Focus on remaining calm.

Procedures (intervention components)
*Prevention methods (supporting component)* Activity content supported by written informational resource, video bank demonstration and home practice. Content includes: Recognising and managing ILO triggers. Laryngeal muscle stretches. Diaphragmatic breathing. Upper airway health regime. *Supporting co‐existing conditions (supporting component)* Activity content supported by written informational resource and home practice. Content includes: When a confirmed co‐existing asthma or airways diagnosis present, teach symptom differentiation in context of ILO management. Support management of any associated co‐morbidities as part of co‐ordinated care. Provide anxiety and stress management techniques, and refer on to psychological services support as clinically indicated. *Bio‐feedback training (supporting component)* Activity content supported by own laryngeal video feedback and home practice review. Content includes: Perform laryngoscopy and provide real time visual feedback in laryngeal movements with and without application of rescue breathing. Expose to identified aggravating triggers with application of techniques to view reversal. *Managing others' reactions to ILO (supporting component)* Activity content supported by written informational resource and home practice. Content includes: Discuss how to manage friends and families' reaction and how they can best support during an ILO episodes. Provide and give support approaches for workplace/social environments. Discuss how to manage stranger reaction. Joint session with individual's nominated other as wished/indicated. *Home practice (cross‐cutting component)* Activity content supported by written informational resource, video bank demonstration and home practice. Content includes: Discuss application of home practice into daily routines (minimum five times daily, 5 min each time; no maximum dose). Focus home practice based on individual need, likely relates to previous session component. Discuss how to integrate into daily routine and provide cues/prompts to support. Support compliance with self‐monitoring strategies.

Who provides
A qualified speech and language therapist, with additional training in laryngeal mechanisms and upper airway disorders should lead U‐ACT intervention delivery. As a guide for competency standard see the ‘*Royal College of Speech and Language Therapists Position paper: The Role of speech and language therapy in upper airway disorders within adult respiratory services (2021); Section 11 Training and education, pages 28–29’* [41]. Intervention providers should familiarise themselves with the supporting U‐ACT handbook (Appendix 10) and complete the U‐ACT intervention training checklist (to satisfaction) prior to delivering the U‐ACT intervention. Members of the multi‐disciplinary team, who have addressed the U‐ACT intervention training checklist (under the supervision of the lead speech and language therapist) and are confident with aspects of competency can deliver those intervention components (e.g., education & empowerment). They can also reinforce supporting components as appropriate to support co‐ordinating an individual's care (e.g., a physiotherapist treating a co‐existing breathing pattern disorder can reinforce the rescue breathing activity).

How: mode of delivery
The U‐ACT intervention is delivered individually, in a 1:1 format. Based on key‐stakeholder evidence, U‐ACT intervention is *not* designed for group therapy. The initial session and bio‐feedback training component occur face‐to‐face. Subsequent sessions can occur face‐to‐face, virtually or over the telephone as determined by individual and service preference. Intervention delivery is communicated and co‐ordinated with other members of the individual's healthcare team.

Where: location
As a minimum, the first intervention session occurs face‐to‐face in the clinic room. The bio‐feedback training component occurs in a clinical environment that is safe and appropriately risk assessed (as per local policy and procedures). Remaining sessions/components occur either face‐to‐face or remotely (over video or telephone call), as preferred by the individual and/or service.

When and how much
The U‐ACT intervention should occur as soon as possible following confirmed diagnosis of ILO. Session 1 (initial face‐to‐face intervention session lasts 45 min) covers the two core components (education & empowerment; reliever breath control). Up to five further 30‐min intervention sessions (sessions 2–6) can occur, with a minimum of two further sessions (sessions 2 & 3), resulting in an overall intervention dosing of between 3 and 6 sessions. All intervention components are covered, with weighting of each component and order of delivery based on individual need. All intervention sessions are delivered in a maximum four‐month period; the schedule is determined by individual/service requirements. Home‐practice cross‐cutting component occurs daily over a minimum of five practice sessions, each lasting at least 5 min in duration. There is no maximum dosing restriction for home practice.

Tailoring
The U‐ACT intervention offers personalisation and tailoring: An intervention titration option enables appropriate dosing, based on individual need. Flexibility for mode of delivery, based on individual and service need Flexibility for choosing weighting and order of delivery of supporting intervention components, based on individual need. The schedule when intervention sessions occur during the four‐month intervention period. Delivery of identified intervention components by other members of the multi‐disciplinary team (see ‘Who provides’ section). In ‘managing others’ reaction’ component, a supportive joint session with identified individuals (e.g., individual's spouse), as appropriate to individual.

## 4 Discussion

We have developed and described a non‐pharmacological intervention for ILO, personalised to the needs and preferences of both those who suffer with the debilitating condition and those who deliver care. Our work has identified the specific components of the intervention, specified the underlying processes that support it, deciphered contextual considerations, detailed mode of delivery, and investigated acceptability. The work received positive stakeholder feedback, supporting intervention design, which is encouraging, but further research to test feasibility and efficacy is needed.

Interventions based on theories of behaviour change have been shown to be more effective [45]. The development of the U‐ACT's intervention is based on specified behavioural change theories, and we are therefore optimistic regarding future efficacy testing. Specifically, the 36 BCTs identified are mapped to theoretical behavioural change theory. This theoretical underpinning informs U‐ACT's causal mechanisms and describes how the U‐ACT intervention could lead to a positive outcome for ILO symptom burden and control, thus reducing the overall impact of the condition. Interestingly, there is synergy with U‐ACT's 36 BCTs and those identified in previous preliminary investigation mapping BCTs to speech therapy non‐pharmacological ILO interventions [22]. Dominant BCT cluster groups include grouping and planning, repetition and substitution, shaping knowledge, feedback and monitoring. In a field with limited evidence, such concurrency in findings is reassuring and further supports the overarching mediators of change identified.

Qualitative analysis of patient perception of the impact of ILO on quality of life identified themes that emphasised (i) the emotional and psychological consequences of ILO and (ii) understanding the importance of ameliorating factors [5]. U‐ACT's COM‐B behavioural diagnosis dominance in psychological capability and reflective motivation addresses these themes. The intervention, by design, aims to strengthen knowledge and psychological skills to engage in necessary mental and reflective processes involving self‐conscious intentions and evaluations. For example, U‐ACT will upskill an individual to be aware of their ILO triggers, facilitate them to have the skills to manage and reduce their ILO symptoms if induced, and provide an education underpinning concrete knowledge that ILO is not life‐threatening (to alleviate perpetuating fears).

Home practice was purposefully allotted as a cross‐cutting theme, based on the weighted importance from Stage 1 and 2 data. Confirmation of the ‘golden thread’ was highlighted when the highest number of BCTs across the components were identified in ‘home‐practice’. However, compliance with home‐practice is a known challenge [46] and tools to support motivation and adherence have been incorporated. Specifically, a video bank resource is detailed, which in other related fields have proven to improve compliance [47].

When considering Mahoney's work exploring factors impacting engagement in speech therapy non‐pharmacological ILO interventions [46], U‐ACT's components align and may, in part, serve to mitigate the risk of non‐adherence. For example, a reported salient theme impacting engagement was social awkwardness. This included difficulty when asking others to change their behaviours to help reduce ILO symptoms. The U‐ACT “managing others' reactions to ILO” component specifically targets how to support and address such social awkwardness. It promotes trying to manage external factors and restructure environmental contexts by utilising the ‘active’ BCT ingredients ‘social support’ and ‘information about others’ approval’.

In a recent randomised controlled trial of speech therapy non‐pharmacological intervention, study investigators terminated the study prior to completion [48]. Although early termination was mainly attributed to complications of placebo delivery, it should be acknowledged that the treatment arm comprised an intervention based on local care. It was not developed robustly to inform a standardised approach and detailed none of the many uncertainties U‐ACT addresses. Despite this, it provides useful insight in how best to approach future evaluation of U‐ACT. As guided by the MRC framework, several perspectives should be considered beyond the binary questions of effectiveness, namely will U‐ACT be implementable, cost effective, scalable, and transferable across contexts [18].

A core element of the MRC framework for developing and evaluating interventions [18] is ‘economic considerations.’ During intervention development, consideration was given during study team meetings informally (which fed into the developed programme theory), but no formal engagement with economic expertise or a cost–benefit analysis occurred. As such, future feasibility work should address pertinent economic questions that matter to decision makers regarding U‐ACT intervention adoption [49].

With regards to home practice dosing, feedback received pointed to a ‘much as possible’ philosophy which provides rationale to the ‘no maximum’ dosing stated. Such an approach could have negative reverse consequence and therefore a minimum dose was set. However, it is acknowledged the minimum does of ×5 times daily, ×5 min duration is an arbitrary unsubstantiated amount as no directive data exists [50].

Outcome reporting was detailed as part of U‐ACTs core component ‘education and empowerment’. However, our Stage 1 systematic review [15] highlights there is no uniform approach in the selected outcomes to monitor treatment effectiveness. We missed opportunity to explore the outcome measures used during the Stage 2 data collection. As such, careful consideration is required when selecting the primary endpoint of any future evaluation of U‐ACT.

Finally, in the present study, the same individuals participated in Stages 2 (primary data collection) and 4 (assessing acceptability) meaning there may have been a selection bias because participants were inherently invested in the intervention under development. Therefore, during any future feasibility testing, it will be important to consider external stakeholder acceptability of U‐ACT to gain insights from those not directly involved in its development.

In conclusion, the U‐ACT intervention is derived from prior practice‐based evidence of ILO treatment and is not an antagonistic approach. However, to the best of our knowledge, this work is the first to apply a well‐established framework to develop a standardised non‐pharmacological intervention for ILO, linked to behavioural change. Importantly, U‐ACT is not entirely prescriptive; it allows for flexibility to support individualised treatment based on clinician judgements of patients' predisposing and precipitating factors.

We are optimistic U‐ACT has a chance of being effective and implementable in practice, as it was developed in co‐design with key stakeholders and is underpinned with its own specific programme theory. However, next steps must include formal feasibility testing (which will include cohesive production of U‐ACT related supporting materials, that is, promotional materials/patient information materials). This will shape a multi‐centre randomised controlled trial, which if effective will provide robust evidence for best practice care.

## Author Contributions

**Jemma Haines:** conceptualization (equal), formal analysis (equal), investigation (lead), methodology (equal), project administration (lead), resources (lead), supervision (equal), visualization (lead), writing – original draft (lead), writing – review and editing (equal). **Jaclyn A. Smith:** conceptualization (equal), formal analysis (equal), investigation (supporting), methodology (equal), resources (supporting), supervision (equal), visualization (supporting), writing – original draft (supporting), writing – review and editing (equal). **Stephen J. Fowler:** conceptualization (equal), formal analysis (equal), investigation (supporting), methodology (equal), resources (supporting), supervision (equal), visualization (supporting), writing – original draft (supporting), writing – review and editing (equal). **Janelle Yorke:** conceptualization (equal), formal analysis (equal), investigation (supporting), methodology (equal), resources (supporting), supervision (equal), visualization (supporting), writing – original draft (supporting), writing – review and editing (equal).

## Ethics Statement

Study approval and registrations were obtained (Northwest Greater Manchester South Research Ethics Committee 24/NW/0010; ISRCTN18291587; PROSPERO CRD42020213187). Informed consent was obtained from all study participants.

## Conflicts of Interest

The authors declare no conflicts of interest.

## Supporting information


**Table S1.** Summarised findings from systematic review.
**Table S2.** Participants and synthesis of identified themes.
**Appendix S1.** The U‐ACT handbook and training checklist guide.

## Data Availability

The data that support the findings of this study are available from the corresponding author upon reasonable request.
