# Supplementary material for: Upper Airway Control Therapy (U‐ACT): The Development of a Non‐Pharmacological Intervention for Inducible Laryngeal Obstruction
Source: Respirology. 2025 Jun 10;30(10):935–48. doi: 10.1111/resp.70073 (PMC12486341; doi:10.1111/resp.70073)
Supplement: Supplementary file 1 — Table S1. Summarised findings from systematic review. Table S2. Participants and synthesis of identified themes. Appendix S1. The U‐ACT handbook and training checklist guide. [file RESP-30-935-s001.docx]

**SUPPORTING INFORMATION**

**Upper Airway Control Therapy (U-ACT): the development of a non-pharmacological intervention for inducible laryngeal obstruction**

Jemma Haines, Jaclyn A Smith, Stephen J Fowler, Janelle Yorke

**CONTENTS**

**Table S1: Summarised findings from systematic review**…………………….Page 2

**Table S2: Participants and synthesis of identified themes**………………….Page 3

**APPENDIX S1: The U-ACT handbook and training checklist guide**…….... Page 5

**Table S1: Summarised findings from systematic review**

| **Included studies** |
| --- |
| - Searching identified 3359 records - Following deduplication, 2308 screened for eligibility - Full text screening in 92 records - 14 studies (n=527 participants) met criteria for inclusion |
| **Methodological quality** |
| - All studies observational by design - Only one presented limited comparison data; no cohort/cross sectional studies - No randomised controlled trials - Joanna Brigges Institute evidence for effectiveness grade = very low level - High risk of bias (in multiple domains) across studies |
| **Interventions** |
| - Inconsistent and incomplete reporting across studies - Treatment duration range 2.5 weeks to 12 months - Heterogeneity in frequency and number of sessions received - Session duration not reported - Only a third provided theoretical basis - 90% of interventions delivered by speech and language therapists - Most frequently reported common components included: supplementary home practice, education, relaxed throat breathing, diaphragmatic breathing, pulsed/focused exhalation, trigger identification and management |
| **Outcomes** |
| - Overall direction of effect positive in 76% measures reported - Outcome reporting weighted on non-validated symptom scales - 59.5% reduction in healthcare utilisation (only reported in three studies) |

**Table S2: Participants and synthesis of identified themes**

| **Participants** | |
| --- | --- |
| - Speech and language therapists: very experienced [median (IQR, range) 19 (13, 6-34) years post qualification and 7(3, 4-25) years of holding a weekly ILO caseload] - Patients: 73% female, middle aged [mean (SD) age 53 (13) years] | |
| **Identified theme** | ***Summary*** |
| Living with ILO | - Overwhelming sense of fear of ILO (particularly of dying) - ILO = negative impact on social activities and employment - Clear demand for better education about ILO (generally in the world; during management; amongst healthcare professionals) |
| Pathway to intervention | - Significant pre diagnosis fears and burden reported - Delayed diagnosis and mismanagement prevalent - Strong opposition to ‘speech and language therapy’ name to describe intervention - Significant motivation for an intervention that aims to give support to control symptoms |
| Care delivery | - Multi-disciplinary care co-ordination identified as crucial - Preferred dosing: 3-6 sessions (lengthier first session), over 3–4-month duration, with mode of delivery flexible - Patients ambivalent who delivered intervention, as long as had appropriate skill set; group intervention not favoured - Strong desire for supporting intervention materials, particularly video resources |
| Intervention components | - Overwhelming support for bio-feedback training as supported understanding significantly - Symptom differentiation beneficial to improve targeted use of asthma medications (when applicable) - Identifying ILO triggers and preventions techniques favoured (releasing baseline laryngeal tension, diaphragmatic breathing, how to achieve healthy laryngeal mucosa) - Techniques to manage escalating and acute ILO symptoms invaluable (remaining calm; nasal inspiration with focused expiration) - Regular home practice the ‘golden thread’ to achieve ILO control, no maximum dosing stipulated |
| Views of intervention | - Patients had belief in non-pharmacological intervention and their capacity to succeed with it - Motivation related to reduced symptoms, relieving anxiety for loved ones and following professional advice - No perceived issues with capability to perform exercises or applying techniques - Strong signals for the need for an optional formal partnership approach (with significant others) to help manage ILO symptoms - Following interventions, patients reported developed controlling skills and a significant reduction in the impact ILO had on them |

**APPENDIX S1: The U-ACT handbook and training checklist guide**

**Upper Airway Control Therapy**

**(U-ACT)**


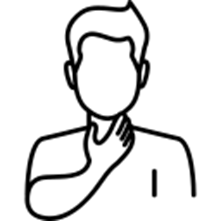


**A non-pharmacological behavioural therapy intervention for adults with inducible laryngeal obstruction (ILO)**

**HANDBOOK**

Version 1, April 2025

ISRCTN18291587


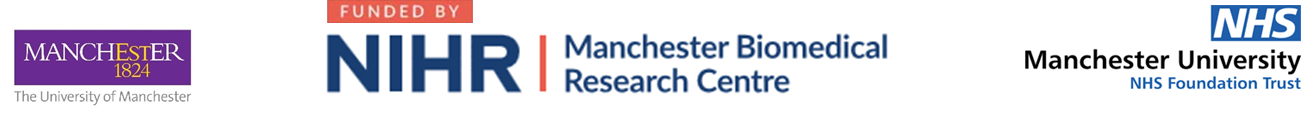


**Contents**

**Preface 3**

**Acknowledgements 3**

**Developing the U-ACT intervention4**

**Theory and mechanisms of action5**

**U-ACT intervention structure 6**

**Delivery of components 7**

**Core components (introduced session 1)8**

**Supporting components 9**

**Home practice cross-cutting component11**

**Dosing and mode of delivery 12**

**Personalisation 13**

**Supporting materials 14**

**U-ACT intervention providers 15**

**Training checklist 16**

**References 17**

**Preface**

Adults with inducible laryngeal obstruction (ILO) suffer significant impact on quality of life and often have high healthcare utilisation (E1); there is frustration amongst healthcare professionals and patients about the lack of understanding for optimal ILO non-pharmacological treatment methods. This handbook summarises the Upper Airway Control Therapy (U-ACT) intervention, which has been developed to offer a standardised approach to treat adults diagnosed with ILO. U-ACT should commence as soon as possible after ILO diagnosis. In its current form, U-ACT is developed and ready for future feasibility testing. This handbook is written to support dissemination of the developed intervention and for the purposes of specifying U-ACT intervention content for research purposes.

**Acknowledgements**

This work has been supported by the National Institute of Health Research Manchester (NIHR) Biomedical Research Centre and the Northwest Lung Centre charity (UK), as part of Jemma Haines’ Doctoral research studies (University of Manchester, UK). Intervention development meeting contributors included Professor Stephen Fowler, Professor Jacky Smith, Mrs Jennifer Butler and Professor Janelle Yorke. Specific thanks go to the healthcare professionals and patients who gave up their time and shared their expertise/lived experiences to shape the development of the U-ACT intervention.

**Developing the U-ACT intervention**

The new MRC framework for developing and evaluating complex interventions (E2) guided intervention development, supported by the INDEX principles for intervention development and design (E3). This ensured key-stakeholder engagement and identification of an underpinning programme theory for the intervention.

The multi-phase research steps were:

1. An evidence review of the current literature (E4)
2. A healthcare professional focus group & semi structured interviews (with individuals diagnosed with ILO) to understand the key considerations and components of the intervention
3. Development of a supporting intervention programme theory to understand the mechanisms of change and support intervention design
4. Assessing acceptability of the intervention protype
5. Description of the final intervention

The TIDieR framework (E5) gave a structure to describe the intervention. Study team development meetings occurred throughout intervention development. Study approval and registrations were obtained as required (Northwest Greater Manchester South Research Ethics Committee 24/NW/0010; ISRCTN18291587; PROSPERO CRD42020213187).

**Theory and mechanisms of action**

The U-ACT intervention comprises 36 behavioural change ‘active ingredient’ techniques. These provide individuals with psychological and physical capability to control ILO, the opportunity to perform techniques in differing environments and strengthened motivation to consciously goal plan, with added self-belief that they can control ILO. U-ACT’s underpinning programme theory is represented in the below causal logic model.


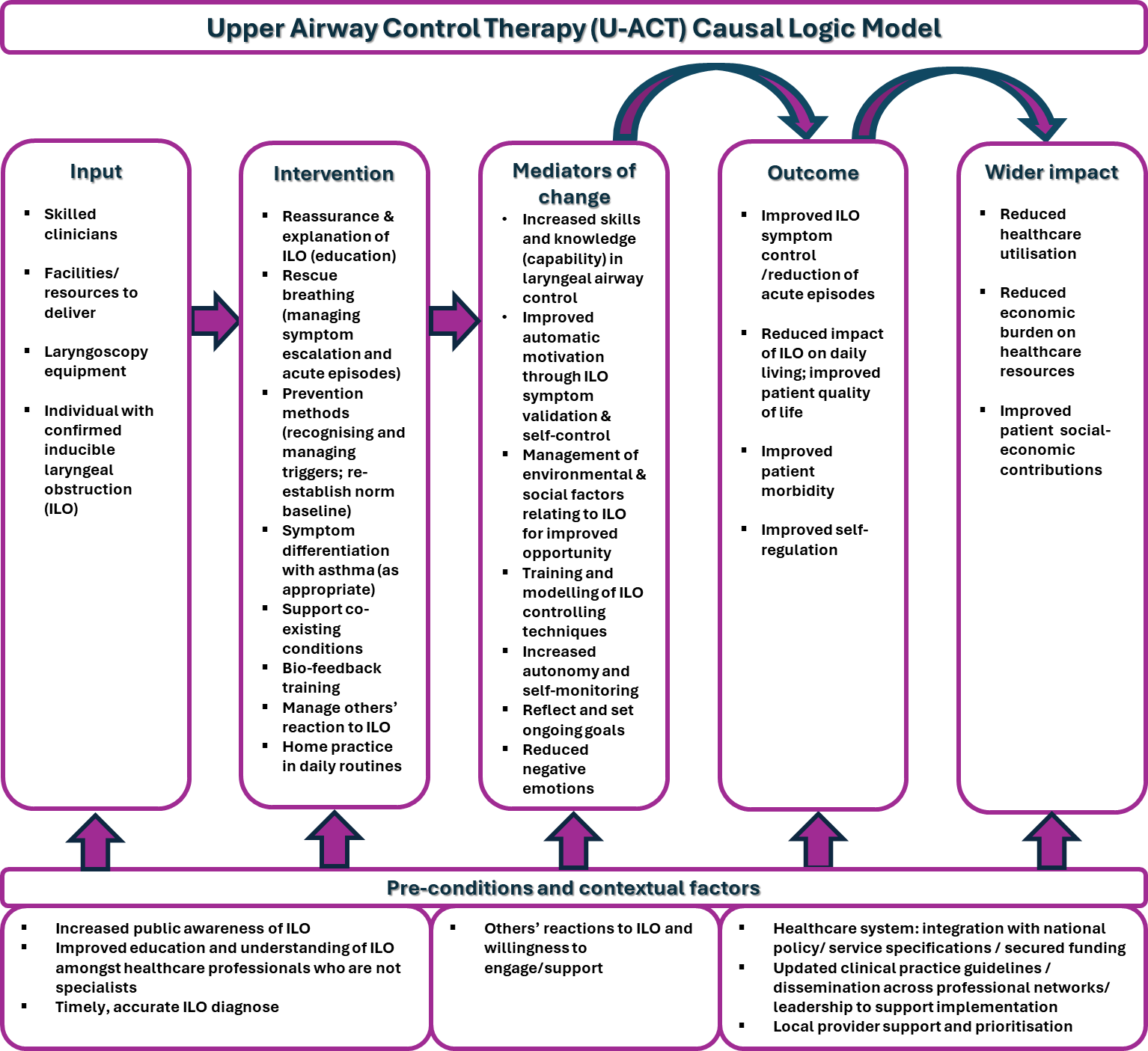


**U-ACT intervention structure**

The U-ACT intervention comprises two core components (education & empowerment; reliver breath control), four supporting components (bio-feedback training; prevention methods; supporting co-existing conditions; managing others’ reactions to ILO) and a cross-cutting home practice component.


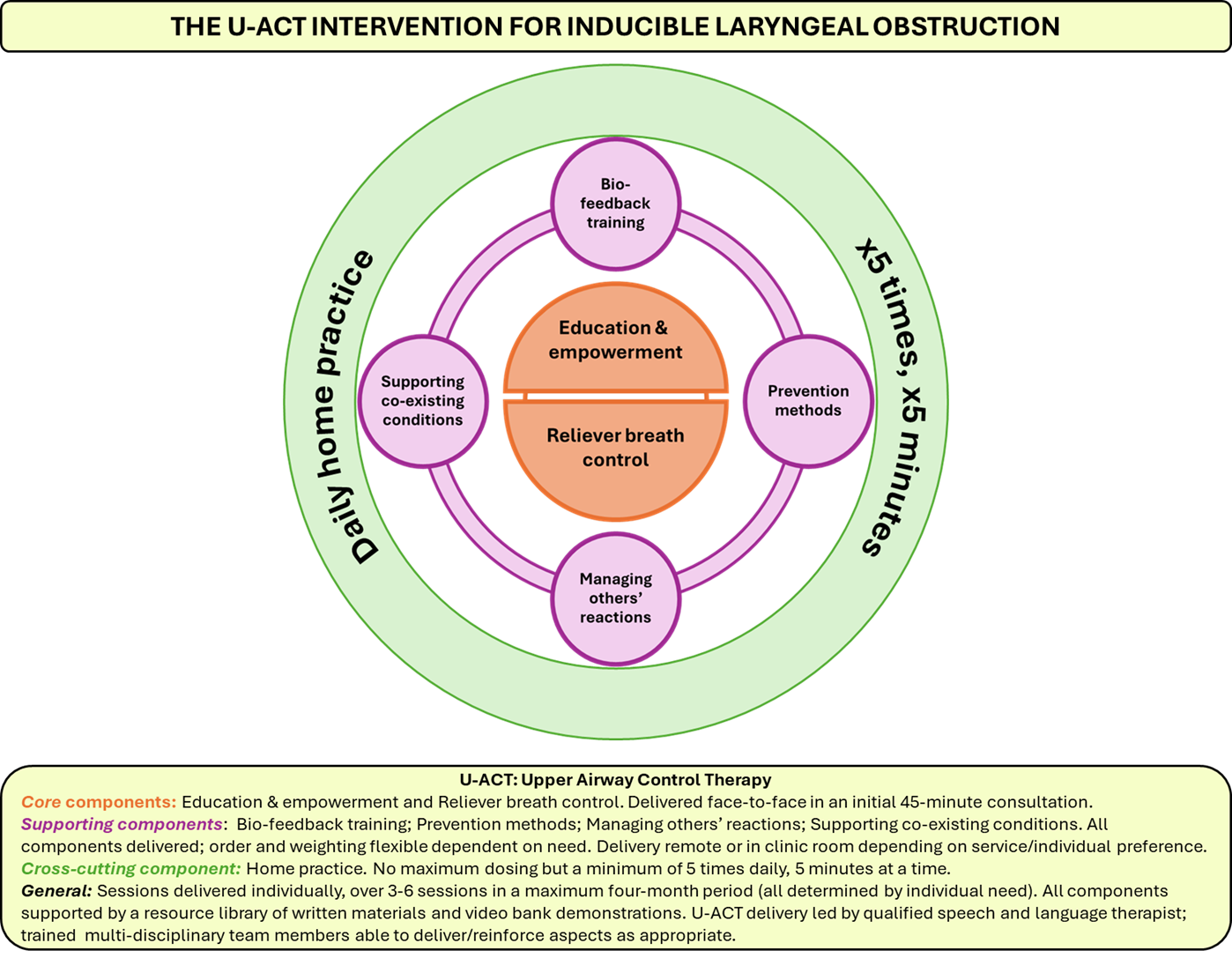


**Delivery of components**

This handbook provides detailed description of U-ACT’s components and related content. However, the specific tasks within the content lists are not prescribed to allow for clinician preference and flexibility. For example, in the *Prevention methods* component ‘diaphragmatic breathing’ is listed content but specific techniques to achieve this are not detailed. In this case, the clinician could utilise Accent Method (E6) foundations, a lying to standing hierarchy focusing on reduced clavicular movement, semi-occluded vocal tract exercise (E7) or any other direct exercise to achieve diaphragmatic control.

All intervention components are delivered. However, flexibility for choosing component weighting and order of delivery is based on individual need (core components must, as a minimum, be covered in the initial intervention session). Some components may require review and reinforcement across several sessions, based on individual need.

**Core components (introduced session 1)**

***Education & empowerment***

Content includes:

- Normal respiration and the larynx
- Laryngeal movements during ILO
- Reassurance and explanation ILO not life-threatening (discuss ‘worst-case’ scenario)
- Current scientific understanding on causes and curability of ILO (in accessible language) i.e. limited to date and therefore focus is on symptom control
- Self-management approaches
- Explain U-ACT intervention development (i.e. informed by needs and experiences of individuals suffering with ILO) and discuss U-ACT structure to set expectations
- Individual goal setting
- Outcome monitoring

Activity content supported by written informational resource and home practice (see supporting materials).

***Reliever breath control***

Content includes:

- Rescue breathing techniques (focus on nasal inspiration and focused expiration, e.g. sniff/blow blow technique)
- Managing ILO escalation through application of rescue breathing
- Focus on remaining calm

Activity content supported by written informational resource, video bank demonstration and home practice (see supporting materials).

**Supporting components (all covered over following intervention sessions)**

***Prevention methods***

Content includes:

- Recognising and managing ILO triggers
- Laryngeal muscle stretches
- Diaphragmatic breathing
- Upper airway health regime

Activity content supported by written informational resource, video bank demonstration and home practice (see supporting materials).

***Supporting co-existing conditions***

Content includes:

- When a confirmed co-existing asthma or airways diagnosis present, teach symptom differentiation in context of ILO management
- Support management of any associated co-morbidities as part of co-ordinated care
- Provide anxiety and stress management techniques, and refer on to psychological services support as clinically indicated

Activity content supported by written informational resource and home practice (see supporting materials).

***Bio-feedback training***

Content includes:

- Perform laryngoscopy and provide real time visual feedback in laryngeal movements with and without application of rescue breathing
- Expose to identified aggravating triggers with application of techniques to view reversal

Activity content supported by own laryngeal video feedback and home practice review (see supporting materials).

***Managing others’ reactions to ILO***

Content includes:

- Discuss how to manage friends and families’ reaction and how they can best support during an ILO episodes
- Provide and give support approaches for workplace/social environments
- Discuss how to manage stranger reaction
- Joint session with individual’s nominated other as wished/indicated

Activity content supported by written informational resource and home practice (see supporting materials).

**Home practice cross-cutting component**

Individuals are encouraged to complete daily home practice for a minimum of five practice sessions, each lasting at least five minutes in duration. There is no maximum dosing restriction for home practice. To support this:

- Discuss application of home practice into daily routines and explore appropriate prompts/cues to facilitate
- Focus home practice based on individual need, likely relates to previous session component
- Discuss compliance with self-monitoring strategies

**Dosing and mode of delivery**

*Dosing*

The U-ACT intervention should occur as soon as possible following confirmed diagnosis of ILO.

- Session 1 (initial face-to-face intervention session lasts 45 minutes) covers the two core components (education & empowerment; reliver breath control).
- Up to five further 30-minute intervention sessions (sessions 2-6) can occur, with a minimum of two further sessions (sessions 2 & 3), resulting in an overall intervention dosing of between 3 and 6 sessions. All intervention components are covered, with weighting of each component and order of delivery based on individual need.
- All intervention sessions are delivered in a maximum four-month period; the schedule is determined by individual/service requirements.

*Mode of delivery*

The U-ACT intervention is delivered individually, in a 1:1 format; U-ACT intervention is *not* designed for group therapy. Intervention delivery is communicated and co-ordinated with other members of the individual’s healthcare team.

The initial intervention session and bio-feedback training component occur face-to-face. Specifically, the bio-feedback training component occurs in a clinical environment that is safe and appropriately risk assessed (as per local policy and procedures).

Subsequent intervention sessions can occur face-to-face, virtually or over the telephone as determined by individual and/or service preference.

**Personalisation**

The U-ACT intervention offers personalisation and tailoring:

- An intervention titration option enables appropriate dosing, based on individual need
- Flexibility for mode of delivery, based on individual and service need
- Flexibility for choosing weighting and order of delivery of supporting intervention components, based on individual need
- The schedule when intervention sessions occur during the four-month intervention period
- Delivery of identified intervention components by other members of the multi-disciplinary team (see U-ACT intervention providers, page 15)
- In ‘managing others’ reaction’ component, a supportive joint session with identified individuals (e.g. individual’s spouse), as appropriate to individual

**Supporting materials**

The U-ACT intervention supporting materials are:

*U-ACT promotional materials*

Information regarding the U-ACT intervention. Aimed for referrer clinicians, patients and associates, the wider multi-disciplinary team and healthcare professionals. Includes general education about ILO. To provide:

- with healthcare provider correspondence following ILO diagnosis
- to individuals when diagnosed with ILO and referred for U-ACT intervention
- as a general educational resource to improve awareness of ILO

*Provider training materials*

This U-ACT handbook provides an information delivery guide and a training checklist for healthcare professionals who will deliver the intervention.

*Patient information materials*

- A written resource to support each of the U-ACT intervention components. Provide these relevant written resources following delivery of a specific component (electronically or paper based depending on individual preference).
- A video bank resource comprising all direct physical exercises included in U-ACT intervention. Videos demonstrate intervention techniques and support the modelling and training function of U-ACT intervention. Provide access following first intervention session.

*Physical materials*

Laryngoscopy imaging equipment with visibly accessible feedback monitors (see procedures, bio-feedback training)

**U-ACT intervention providers**

A qualified speech and language therapist, with additional training in laryngeal mechanisms and upper airway disorders, should lead U-ACT intervention delivery. As a guide for competency standard see the ‘*Royal College of Speech and Language Therapists Position paper: The Role of speech and language therapy in upper airway disorders within adult respiratory services (2021); Section 11 Training and education, pages 28-29’* (E8).

Intervention providers should familiarise themselves with this U-ACT handbook and complete the U-ACT intervention training checklist (page 16) prior to delivering the U-ACT intervention.

Members of the multi-disciplinary team, who have addressed the U-ACT intervention training checklist (under the supervision of the lead speech and language therapist) and are confident with aspects of competency can deliver those intervention components (e.g. education & empowerment). They can also reinforce supporting components as appropriate to support co-ordinating an individual’s care (e.g. a physiotherapist treating a co-existing breathing patter disorder can reinforce the rescue breathing activity).

**Training checklist**

To lead delivery of U-ACT intervention, as a minimum, the following checklist should be adhered to:

- Qualified speech and language therapist, compliant with regulatory body competency/requirements and working within their scope of practice
- Knowledge, understanding and ability to deliver a wide range of therapeutic interventions for upper airway disorders
- Counselling skills and experience in supporting behavioural change
- Understanding of complementary roles of the multi-disciplinary team involved in the management of ILO
- Ability to interpret (and ideally perform) continuous laryngoscopy to provide bio-visual feedback and have the skills for real-time, responsive, teaching

For multi-disciplinary team members who may support aspects of U-ACT intervention delivery, as a minimum, the following checklist should be adhered to:

- Member of the multi-disciplinary care team of the individual receiving U-ACT intervention
- Identify, and have contact with the lead speech and language therapist responsible for the overall delivery of U-ACT (i.e. the lead U-ACT provider)
- Receive additional training, as identified and required, to support the aspect of U-ACT intervention delivery planned (supported by lead U-ACT provider)
- For each individual case, ensure co-ordination of care with the lead U-ACT provider

**References**

E1. Traister RS, Fajt ML, Petrov AA. The morbidity and cost of vocal cord dysfunction misdiagnosed as asthma. Allergy Asthma Proc. 2016;37(2):25-31.

E2. Skivington K, Matthews L, Simpson SA, Craig P, Baird J, Blazeby JM, et al. A new framework for developing and evaluating complex interventions: update of Medical Research Council guidance. Bmj. 2021;374:n2061.

E3. O'Cathain A, Croot L, Duncan E, Rousseau N, Sworn K, Turner KM, et al. Guidance on how to develop complex interventions to improve health and healthcare. BMJ Open. 2019;9(8):e029954.

E4. Haines J, Smith JA, Wingfield-Digby J, King J, Yorke J, Fowler SJ. Systematic review of the effectiveness of non-pharmacological interventions used to treat adults with inducible laryngeal obstruction. BMJ Open Respir Res. 2022;9(1):e001199.

E5. Hoffmann TC, Glasziou PP, Boutron I, Milne R, Perera R, Moher D, et al. Better reporting of interventions: template for intervention description and replication (TIDieR) checklist and guide. Bmj. 2014;348:g1687.

E6. Thyme-Frøkjær K, Frøkjær-Jensen B. The Accent Method. Oxford, UK: Compton Publishing 2001.

E7. Meerschman I, D'Haeseleer E, Kissel I, De Vriese C, Tomassen P, Dochy F, et al. Immediate effects of straw phonation in air or water on the laryngeal function and configuration of female speech-language pathology students visualised with strobovideolaryngoscopy: A randomised controlled trial. Int J Lang Commun Disord. 2023;58(3):944-58.

E8. Haines J, Esposito K, Murphy J, Pargeter N, Selby J, Slinger C, et al. The role of speech and language therapy in upper airway disorders within adult respiratory services. Position Paper. London Royal College of Speech and Language Therapists 2021.
